# Mediating pathways of neuroticism and social anxiety in the relationship between childhood trauma and the fear of missing out among Chinese college students

**DOI:** 10.3389/fpsyt.2022.933281

**Published:** 2022-08-23

**Authors:** Jiale Shi, Wei Li, Chengwen Han, Jingying Han, Fang Pan

**Affiliations:** ^1^Department of Second Clinical Medical School, Cheeloo College of Medicine, Shandong University, Jinan, China; ^2^Department of Basic Medical School, Cheeloo College of Medicine, Shandong University, Jinan, China; ^3^Department of Medical Psychology and Ethics, School of Basic Medical Sciences, Cheeloo College of Medicine, Shandong University, Jinan, China

**Keywords:** childhood trauma, neuroticism, social anxiety, fear of missing out, college students

## Abstract

Recent research has identified various risk factors for fear of missing out. However, studies on the potential influence of childhood trauma on the fear of missing out remain scarce, and little is known regarding the mediating mechanisms underlying this relationship. In this study, we examine the predictive role of childhood trauma on the fear of missing out among college students and investigate whether neuroticism and social anxiety mediate the relationship between childhood trauma and the fear of missing out. A sample of 1,266 Chinese college students completed questionnaires regarding childhood trauma, neuroticism, social anxiety, and the fear of missing out. The results indicated that (a) childhood trauma is positively associated with the fear of missing out, (b) both neuroticism and social anxiety mediate the relationship between childhood trauma and the fear of missing out, and (c) neuroticism and social anxiety sequentially mediate the relationship between childhood trauma and the fear of missing out. These findings have crucial implications for the prevention and intervention of the fear of missing out among college students.

## Introduction

Smartphones have become an inseparable part of life (1). More than 36% of people worldwide are smartphone users (Statista: Number of smartphone users worldwide from 2014 to 2020). Further, in China, smartphone users comprise 99.7% of the entire Chinese population. According to the 47th Statistical Report on Internet Development in China, as of December 2020, smartphone internet users in China reached 989 million. Owing to the increase in the time spent and the frequency of accessing social media through smartphones, users may worry about or fear missing information posted by others as well as responses to their messages. This attribute results in the failure to benefit from social media as well as in negative emotions, such as anxiety and worry. This state of smartphone users is called the fear of missing out (FOMO) (2). Previous studies have demonstrated that a high degree of FOMO among college students has a negative effect on the state of their studies (3, 4). College students with high levels of FOMO can be characterized as those afraid of missing important information. In the long term, such individuals rely excessively on social media and can even develop addictive tendencies. Such tendencies result in adverse effects on the mental health of college students (5). Moreover, distracted driving and higher alcohol consumption, which are unsafe behaviors, may be associated with high levels of FOMO among college students (6, 7).

Recent studies have proven that psychological factors, such as personality traits and psychological needs, are closely related to FOMO (8). High levels of neuroticism and social anxiety have positive predictive effects on the levels of FOMO among college students (9–11). Liftiah reports that FOMO among college students was associated with neuroticism, extraversion, and agreeableness and not with conscientiousness and openness (12). Blackwell finds that neuroticism can predict FOMO, but extraversion cannot (8). Sabah believes that high levels of neuroticism among individuals have positive predictive effects on their FOMO (9). Jiang reports that neuroticism, extraversion, and conscientiousness have significant associations with FOMO, whereas agreeableness and openness do not (13). It seems that neuroticism is more strongly associated with FOMO than other personality dimensions; thus, we focus on neuroticism. Moreover, individuals suffering from social anxiety are susceptible to problems in interpersonal relationships and find it significantly difficult to interact directly with people in face-to-face contexts (14, 15). Such individuals use mobile phone calls or text messages to reduce interpersonal anxiety, and this approach provides them with an elevated sense of control over communication (14). If their feelings of anxiety are not regulated, such individuals will spend additional time on their phones and become dependent. Additionally, individuals that overuse mobile phones have a higher sense of social isolation (16). Notably, a positive correlation exists between social anxiety and smartphone overuse (17). Social anxiety mediates the relationship between anxiety and smartphone addiction (11). Further, significant positive correlations exist among neuroticism, social anxiety, and FOMO (11).

Childhood trauma results from acts of abuse or neglect, often committed by parents or caregivers, and are likely to “harm or threaten a child” (18). Child abuse involves various types of physical, emotional, and sexual abuse as well as physical and emotional neglect. According to a worldwide meta-analysis of randomized controlled trials associated with the prevention of child maltreatment, the prevalence levels of sexual abuse, physical neglect, emotional neglect, physical abuse, and emotional abuse were estimated at 12.7, 16.3, 18.4, 22.6, and 36.3%, respectively (19). According to other studies on childhood experiences and trauma, 45.5% of Chinese college students reported unpleasant childhood experiences (20). People who experience emotional trauma exhibit tendencies of hopelessness, low self-esteem, reduced feelings of social support, and poor satisfaction with life (21). Bock et al. find that young people with trauma are more likely to develop negative emotions, though they did not distinguish among the types of trauma in their study (22). This suggests that such individuals may find it challenging to identify and express their emotions, which can result in interpersonal problems among individuals who have experienced emotional or physical trauma. Meanwhile, the versatility, portability, and timeliness of mobile phone-based communication can satisfy these individuals’ need for communication with their peers (23), resulting in their excessive reliance on social media, which may further contribute to their FOMO. Moreover, recent studies have demonstrated that although mobile phone addiction is associated with FOMO, emotional abuse and emotional neglect during childhood are associated with addiction to the Internet (24), and physical and emotional neglect have a prominent positive predictive effect on mobile phone addiction (25). Such studies show that both physical and emotional trauma can predict problematic usage of the Internet or phones, indicating that a relationship between childhood trauma and FOMO may exist. To the best of our knowledge, studies that investigate the exact relationship between these factors are yet to be conducted.

Various studies have established that childhood trauma among college students is significantly and positively correlated with social anxiety (10, 26). It is well known that childhood trauma has a significant impact on personality (22, 26, 27). People who experience childhood trauma tend to exhibit neuroticism (28–32), and individuals with neurotic personalities are highly prone to social anxiety (19). However, the relationship between childhood trauma and FOMO as well as the role of neuroticism and social anxiety with regard to childhood trauma and FOMO are yet to be clarified.

In this study, we hypothesize that (a) childhood trauma is positively correlated with FOMO, (b) neuroticism and social anxiety play mediating roles between childhood trauma and FOMO, and (c) neuroticism and social anxiety moderate the relationship between childhood trauma and FOMO sequentially.

## Materials and methods

### Participants and procedures

We conducted a cross-sectional study through an online survey between July 25 and August 1, 2021. For this survey, we randomly selected one campus among the eight campuses of Shandong University, after which we selected 24 classes on this campus through the random sampling method. To facilitate unified management, all of the selected classes had class-specific WeChat groups, which included all of the enrolled undergraduate students. All of the participants provided informed consent online, and they were informed about the purpose of the study in accordance with Chinese legislation. The study was approved by the Ethics Committee of the School of Basic Medical Sciences of Shandong University [No. ECSBMSSDU 2021-1-096].

The applet with the questionnaires, including the Social Media FOMO Scale, the brief version of the Childhood Trauma Questionnaire, the Interaction Anxiousness Scale, and the Big Five Inventory, was delivered to the undergraduates in their WeChat groups. The participants who volunteered to take the online survey and completed the questionnaire received compensation for research assistance. A total of 1,400 questionnaires were sent out, and 1,266 questionnaires were valid. Among them, 540 (42.7%) were completed by males, and 726 (57.3%) were completed by females.

### Measures

#### Fear of missing out

The Social Media FOMO Scale (Chinese version) compiled by Zhao et al. (33) was used to measure FOMO levels. The questionnaire comprises 17 items measuring four dimensions: psychological motivation, cognitive motivation, behavioral performance, and emotional dependence. The items were rated using a 5-point Likert scale (1 = disagree completely, 5 = completely agree). The higher the total score, the higher levels of the FOMO. The internal consistency coefficient of the questionnaire used in this study was 0.979.

#### Childhood trauma

The brief Chinese version of the Childhood Trauma Questionnaire (CTQ-SF) compiled by Zhao et al. (34) was used to evaluate individuals’ childhood trauma experiences. The scale comprises 28 items, including 25 clinical and three validity items. This scale is divided into five subscales: emotional abuse, physical abuse, sexual abuse, emotional neglect, and physical neglect. The entire scale was used to analyze trauma level in the present study. Fu and Yao (35) verified the reliability and validity of the scale using college students as the subjects; the results showed that the scale had good reliability and validity, and it had specific applicability in the context of Chinese cultural backgrounds. In this study, the internal consistency coefficient of the questionnaire was 0.856.

#### Social anxiety

The Chinese version of the Interaction Anxiousness Scale (IAS) (36, 37) was used to determine levels of social anxiety. The scale comprises 15 items rated using a 5-point scale (1 = disagree completely, 5 = completely agree). Higher scores indicated higher levels of social anxiety. The scale has a satisfactory measurement index and is suitable for Chinese college students. In this study, the internal consistency coefficient of the questionnaire was 0.969.

#### Personality

The Chinese version of the Big Five Inventory (BFI) (38–40) was used to evaluate the personality traits of college students. The scale comprises 60 items, including five dimensions: neuroticism, extraversion, openness, conscientiousness, and agreeableness. The scale was rated using a 5-point scale (1 = disagree completely, 5 = completely agree). Only neuroticism was analyzed in the present study. The coefficients of internal consistency for each sub-questionnaire used in this study were 0.757, 0.761, 0.698, 0.862, and 0.976.

## Results

### Correlation between childhood trauma, neurotic personality, social anxiety, and the fear of missing out

Pearson correlations between the variables are shown in Table 1. First, childhood trauma is positively correlated with neuroticism and social anxiety and negatively correlated with openness, extraversion, agreeableness, and conscientiousness. Second, neuroticism is positively correlated with social anxiety, whereas openness, extraversion, agreeableness, and conscientiousness are negatively correlated. Third, neuroticism, social anxiety, and FOMO demonstrated a positive correlation. In view of the high correlation between neuroticism and social anxiety (*r* = 0.913, *P* < 0.01) and between neuroticism and FOMO (*r* = 0.855, *P* < 0.01), neuroticism is likely more representative than other dimensions in the BFI used in this study, which is consistent with the content related to personality mentioned in the introduction. Therefore, other dimensions are not discussed further.

**TABLE 1:** Correlation between childhood trauma, neuroticism, social anxiety, and the fear of missing out (FOMO).

Variables	$\bar{x}\pm \text{SD}$	1	2	3	4	5	6	7	8
1 Childhood trauma	37.49 ± 10.21	1							
2 Neuroticism	37.43 ± 16.58	0.550**	1						
3 Openness	44.35 ± 6.64	−0.305**	−0.110**	1					
4 Extraversion	42.78 ± 6.71	−0.265**	–0.019	0.661**	1				
5 Agreeableness	44.23 ± 6.59	−0.316**	−0.158**	0.630**	0.523**	1			
6 Conscientiousness	47.38 ± 7.58	−0.348**	0.010	0.671**	0.622**	0.689**	1		
7 Social anxiety	48.98 ± 17.50	0.526**	0.913**	−0.250**	−0.175**	−0.280**	−0.122**	1	
8 FOMO	53.53 ± 19.75	0.526**	0.855**	−0.125**	0.008	−0.202**	−0.095**	0.871**	1

**p < 0.01.

### Stepwise regression analysis

We performed stepwise regression analysis, with FOMO as the dependent variable and childhood trauma, neuroticism, and social anxiety as the predictive variables. First, only childhood trauma was used as the predictive variable (Model 1). Neuroticism and social anxiety were placed in the second layer (Models 2 and 3). Neuroticism was placed in the second layer, and social anxiety was placed in the third layer (Model 4). The results are listed in Table 2.

**TABLE 2:** Results of stepwise regression analysis.

Model	Dependent variable	Predictive variable	β	*t*	*R* ^2^	*P*
1	FOMO	Constant		8.560		<0.001
		Childhood trauma	0.526	21.984	0.276	<0.001
2	FOMO	Constant		10.607		<0.001
		Childhood trauma	0.080	4.610		<0.001
		Neuroticism	0.811	46.885	0.736	<0.001
3	FOMO	Constant		1.248		0.212
		Childhood trauma	0.093	5.821		<0.001
		Social anxiety	0.822	51.253	0.765	<0.001
4	FOMO	Constant		4.371		<0.001
		Childhood trauma	0.061	3.894		<0.001
		Neuroticism	0.335	10.198		<0.001
		Social anxiety	0.533	16.528	0.782	<0.001

FOMO, fear of missing out.

In Model 1, childhood trauma significantly predicted FOMO (β = 0.526, *P* < 0.001). Therefore, Hypothesis (a) was supported. The variables in Model 4 accounted for 78.2% of FOMO, better than the variables in Model 1 (27.6%), Model 2 (73.6%), and Model 3 (76.5%). The performance levels of Models 2, 3, and 4 were confirmed through stepwise regression analysis, but the mediation effect needs to be verified further.

### Mediation analysis

#### Mediating roles of neuroticism and social anxiety in the relationship between childhood trauma and the fear of missing out

We used AMOS24.0 to test the mediating role of neuroticism in the relationship between childhood trauma and FOMO. Bootstrapping was applied to verify the mediation effect, as shown in Table 3. We established that the indirect and direct effects were significant, indicating that the mediation effect was partial rather than complete. Therefore, neuroticism partially mediates the relationship between childhood trauma and FOMO (indirect effect = 0.446, SE = 0.033, 95% CI = [0.409, 0.482]). Mediating effects accounted for 84.79% of the total effect of neuroticism and FOMO.

**TABLE 3:** Pathways of the mediation model using bootstrapping.

Model	Effect type	Pathway	Effect	95% CI lower	95% CI upper
2	Direct	Childhood trauma→neuroticism	0.550**	0.507	0.591
		Neuroticism→FOMO	0.811***	0.781	0.840
		Childhood trauma→FOMO	0.080**	0.039	0.121
	Indirect	Childhood trauma→neuroticism→FOMO	0.446***	0.409	0.482
3	Direct	Childhood trauma→social anxiety	0.526**	0.479	0.574
		Social anxiety→FOMO	0.822***	0.789	0.851
		Childhood trauma→FOMO	0.093***	0.058	0.130
	Indirect	Childhood trauma→social anxiety→FOMO	0.433***	0.392	0.473
4	Direct	Childhood trauma→neuroticism	0.550**	0.507	0.591
		Neuroticism→social anxiety	0.913**	0.902	0.923
		Social anxiety→FOMO	0.824***	0.791	0.852
		Childhood trauma→FOMO	0.094***	0.058	0.130
	Indirect	Childhood trauma→neuroticism→social anxiety→FOMO	0.413***	0.377	0.449

FOMO, fear of missing out. **p < 0.01; ***p < 0.001.

Similar procedures were conducted to evaluate the mediating role of social anxiety in the relationship between childhood trauma and FOMO. Consequently, social anxiety was found to partially mediate the relationship between childhood trauma and FOMO (indirect effect = 0.413, SE = 0.031, 95% CI = [0.392, 0.473]). The mediation effect accounted for 82.32% of the total effect of social anxiety and FOMO. Therefore, Hypothesis (b) was supported.

#### Examining the multiple mediation model

**FIGURE 1:**
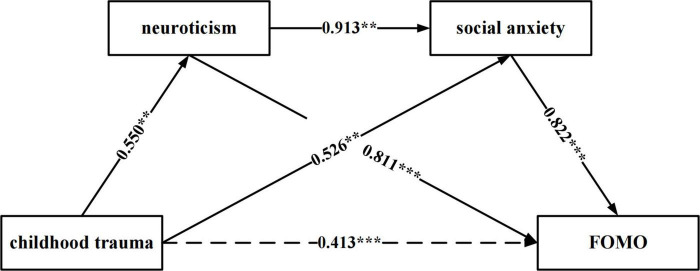
The multiple mediation model. The link between childhood trauma and FOMO is mediated by neuroticism and social anxiety. Path values are the path coefficients. **p < 0.01; ***p < 0.001.

As shown in Figure 1 and Table 3, all pathways were significant. The sequential pathway of “childhood trauma → neuroticism → social anxiety → FOMO” was significant (indirect effect = 0.413, SE = 0.030, 95% CI = [0.377, 0.449]). This multiple mediation model accounted for a significant amount of variance regarding FOMO levels among college students (total effect = 0.507). Therefore, Hypothesis (c) was supported.

In conclusion, all three hypotheses were supported; the results are as follows: (a) childhood trauma is positively associated with FOMO, (b) both neuroticism and social anxiety mediate the relationship between childhood trauma and FOMO, and (c) neuroticism and social anxiety sequentially mediate the relationship between childhood trauma and FOMO.

## Discussion

In this study, we explored the predictive role of childhood trauma on FOMO and the mediating role of neuroticism and social media in this relationship with a sample of students from a Chinese college. The results showed that childhood trauma positively predicted FOMO, and this relationship was sequentially mediated by neuroticism and social anxiety.

Consistent with our hypotheses, this study showed that neuroticism, a personality trait, plays a mediating role in the relationship between childhood trauma and FOMO among college students. In other words, childhood trauma can be used to predict neuroticism, which, in turn, facilitates FOMO among college students. Therefore, neuroticism is an outcome affected by childhood trauma, and it is also an internal motivation for FOMO. The results of our investigation corroborate the conclusions of previous studies proving the existence of significant positive correlations between childhood trauma and personality traits. Additionally, various types of childhood trauma may have different effects on the development of personality dimensions (19). Moreover, previous studies have demonstrated that childhood trauma is positively associated with neuroticism (21), and neuroticism positively affects FOMO (20). In this study, significant positive correlations among childhood trauma, neuroticism, and FOMO were established, corresponding with studies conducted by Kumari and Wan. Additionally, neuroticism is a mediator in the relationship between childhood trauma and FOMO among college students.

Similarly, social anxiety is an outcome affected by childhood trauma, and it is also an internal motivation for FOMO. Previous studies have proven that children who suffer from negative childhood experiences often fail to form secure attachments and suffer from high levels of social anxiety throughout adulthood (20). Social anxiety levels can predict FOMO among such individuals (11). In this study, we examined the relationship between childhood trauma, social anxiety, and FOMO simultaneously, and we primarily focused on the mediating role of social anxiety. Consistent with our hypotheses, social anxiety is a mediator in the relationship between childhood trauma and FOMO among college students.

Finally, the results showed that the role of neuroticism and social anxiety in mediating the association between childhood trauma and FOMO among college students is parallel and sequential. Previous studies have reported that social anxiety acts as a mediator between neuroticism and FOMO among teenagers and college students (11). In this study, through the multiple mediation model (Model 4), we further determined a positive correlation between neuroticism and social anxiety, and we established that neuroticism and social anxiety play mediating roles sequentially. Our findings support the notion that childhood trauma affects multiple psychological functions throughout life, including personality traits (41–43). Neurotic individuals often display obvious emotional traits, such as suspicion (44), sensitivity (26), and anger (22), which induce social interaction problems, such as social anxiety. As a result of neuroticism and social anxiety among such individuals, online communication through mobile phone-based social media applications becomes an alternative to face-to-face communication and provides opportunities for improved expression and a heightened sense of communication control (45). In the long run, the communication employed by such individuals through social media replaces offline communication. Moreover, because of their sensitive, neurotic personalities, they pay significant attention to messages, and eventually, their FOMO is manifested.

In conclusion, through a multiple mediation model, this study provides a complex understanding of the way in which childhood trauma affects FOMO. We established three mediating pathways of neuroticism and social anxiety in the relationship between childhood trauma and FOMO among Chinese college students. Moreover, neuroticism and social anxiety sequentially mediate the relationship between childhood trauma and FOMO.

This study has limitations that must be considered when interpreting the results. First, the data were obtained through self-assessment *via* online surveys, which means that some deviations must exist. Through Harman’s single-factor test, it can be concluded that the surveys are not significantly affected by the deviation of standard methods. Therefore, further studies using clinical interviews are required to ensure a highly comprehensive assessment. Second, causal reasoning was limited because of the cross-sectional data used in this study. In our future studies, we will use a longitudinal design to evaluate the performance of the multiple mediation model used in this study. Third, some detailed mechanisms that connect childhood trauma to FOMO remain unclear. Such mechanisms include the shortest path from a specific subtype of trauma to a specific subtype of FOMO.

## Data availability statement

The original contributions presented in this study are included in the article/supplementary material, further inquiries can be directed to the corresponding authors.

## Ethics statement

The studies involving human participants were reviewed and approved by the Ethics Committee of the School of Basic Medical Sciences of Shandong University (No. ECSBMSSDU 2021-1-096). The patients/participants provided their written informed consent to participate in this study.

## Author contributions

JS: conceptualization, data curation, and writing—original draft preparation. WL: conceptualization, formal analysis, and writing—original draft preparation. FP: validation, project administration, and writing—reviewing and editing. CH and JH: investigation. All authors contributed to the article and approved the submitted version.
